# Small Cell Carcinoma of the Ovary of Hypercalcemic Type: A Case Report

**DOI:** 10.1155/2012/461873

**Published:** 2012-12-30

**Authors:** S. Zaied, O. Gharbi, A. Zayène, R. Rjiba, R. Hadhri, A. Hadded, M. Hochlef, L. Ben Fatma, S. Ben Ahmed

**Affiliations:** ^1^Department of Oncological Medicine, CHU Farhat Hached, 4000 Sousse, Tunisia; ^2^Service of Pathology, CHU Fattouma Bourguiba, 5000 Monastir, Tunisia; ^3^Department of Gynecology and Obstetrics, Hospital Fattouma Bourguiba, 5000 Monastir, Tunisia

## Abstract

*Introduction.* The small cell carcinoma of hypercalcemic type of ovary is a very aggressive tumor. It is associated with two-thirds of cases with hypercalcemia most often asymptomatic. It occurs mostly for young women. The treatment combines surgery, chemotherapy, and radiotherapy. *Case Presentation.* We report a case of small cell carcinoma of the ovary hypercalcemic type in a young Tunisian woman aged 25 years after a severe abdominal pain syndrome and a large ovarian mass discovered in scanner; a laparotomy was performed by radical surgery. The pathological examination of the specimen confirmed the diagnosis. The radiological assessment performed after surgery showed a continuing evolution. Palliative chemotherapy was established, and the patient had died two months after diagnosis. *Conclusion.* The hypercalcemic small cell carcinoma of the ovary is a rare disease of poor prognosis.

## 1. Introduction

The small cell carcinoma of the ovary is a rare tumor and of very modest prognosis. It usually occurs for young women and partners in two-thirds of which were described in the literature of paraneoplastic hypercalcemia.

We report a new case of small cell carcinoma of hypercalcemic type of the ovary for a young woman whose evolution was rapidly mortal with early recurrence after an optimal surgery.

## 2. Case Presentation

Tunisian patient, 25, nulliparous without previous history with the disease, presented in June 2009 with an acute abdominal pain syndrome.

Clinical examination revealed a large pelvic mass measuring 20 × 18 cm compared to 2 fixed plans. Biology revealed an inflammatory syndrome with accelerated versus, a high CRP. Serum calcium was normal at 2.29 mmol/L (normal: 2.25 mmol/L-2, 6 mmol/L). The Ag CA125 was high at 186.8 IU/mL (5 × normal).

The abdominopelvic scanner objectified a voluminous abdominopelvic cystic and fleshy mass measuring 24 × 18 × 15 cm that grows at the expense of the left ovary. That was associated to a peritoneal outpouring from a medium abundance of perihepatic, perisplenic, and in paracolic gutters right and left with a velvety aspect of the parietal peritoneum and a discrete expansion of bilateral renal cavities.

**Figure 1:**
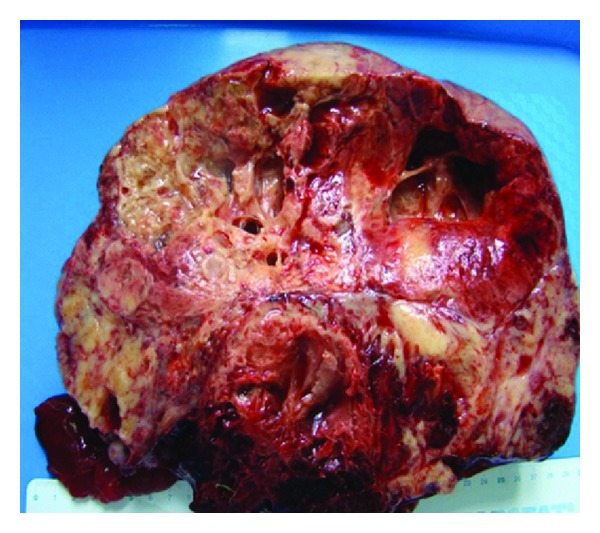
Macroscopic appearance of the cystic and solid mass with necrotic and hemorrhagic aspect.

An exploratory laparotomy was done and found an irregular and variable solid and cystic tumor vascular measuring 40 × 35 cm at the expense of the left ovary; right ovary was normal. Hysterectomy with bilateral salpingooophorectomy and peritoneal samples were made. During the macroscopic examination, it was a mass of 29 cm, solid and cystic, with the site of significant alterations that are necrotic and hemorrhagic (Figure 1).

**Figure 2:**
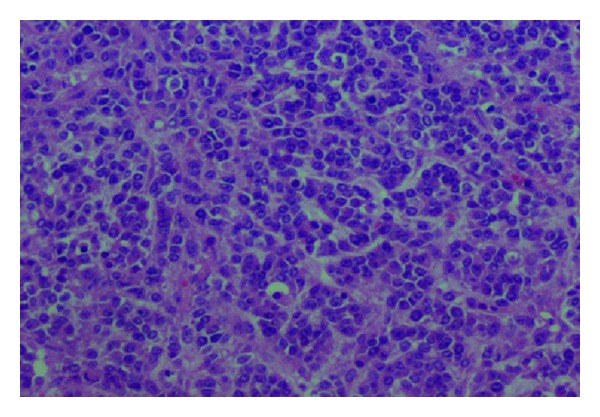
Monomorphic round cells with many mitoses (×200).

Histological examination showed a diffuse proliferation of small round cells, monomorphic, with numerous mitoses (Figure 2). The cells had a different appearance in places; they were larger, with abundant cytoplasm and clear nuclei; nucleoli.

In the immunohistochemical study, the cells expressed vimentin and EMA. CD34 marked cells that are large. The cells were negative with inhibin antibody, cytokeratin, and LCA.

All these elements raised the diagnosis of small cell carcinoma of hypercalcemic type of the left ovary. The peritoneal fluid cytology was positive, and the tumor was classified as stage IC. On the third postoperative day, the patient had presented an acute respiratory distress syndrome caused by infection compounded by bilateral pleural effusion requiring a brief hospitalization in intensive care, despite of being curbed by antibiotics and pleural drainage.

Abdominopelvic ultrasound performed six weeks after surgery showed an abdominopelvic heterogeneous large tissue mass measuring 13 × 11 cm associated with a partitioned intraperitoneal effusion of low abundance and intestinal distension and peritoneal masses joins in remote the most voluminous seat at the right iliac fossa measuring 5 cm of diameter.

Serum calcium was normal and the Ag CA125 was rising (8 × normal). Palliative chemotherapy according to the BEP regimen (Etoposide: 100 mg/m^2^ d1 to d5, Cisplatyl: 20 mg/m^2^ d1 to d5, bleomycin 30U d2, d9, d16) was established. The patient had received only one treatment and passed away in an array of severe sepsis. The decline is two months from diagnosis.

## 3. Discussion

The small cell carcinoma of hypercalcemic type of the ovary is a rare tumor seen mainly in young women. Its first description was recently published in 1982 by Dickersin et al. [1]. Less than 250 cases have been reported in the literature [1, 2].

The average age of onset of these tumors is 24 years [3]; our patient was 25 years old at diagnosis. The clinical signs are nonspecific: abdominal pain and/or abdominopelvic pain, abdominal distension, weight loss, nausea, vomiting, and anorexia [1, 3].

In two-thirds of cases, this tumor is associated with hypercalcemia; our patient had a normal serum calcium.

The physiopathologie of hypercalcemia is unclear. In some cases it may be related to direct the secretion of PTH by tumor-RP (parathyroid hormone-related protein) detected by immunohistochemistry [4].

The largest series, reported by Young, includes 150 patients with small cell carcinoma. This is most often a bilateral tumor of significant size between 6 cm and 26 cm, with an average of 15 cm [3]; in our case, the size of the mass was 24 × 18 × 15 cm. There is an extra ovarian abdominal dissemination in half of the cases. Indeed, Young reported that 50% of tumors were stage I FIGO classification, two-thirds are stage IA, and one-third are IC stage, while over 45% are diagnosed at FIGO stage III and only 5% are divided into stages II and IV [3].

The histological diagnosis is problematic because of similarities with other ovarian tumors. 50% of cases are described next to the small cells, larger cells with large nuclei, and abundant cytoplasm [3]. These large cells can sometimes be the main contingent and form a histological variant of small cell carcinoma.

The differential diagnosis is primarily with the tumor granulosa cell type but also with the juvenile cell tumor of the adult-type granulosa, lymphoma, small cell lung carcinoma, and metastases of melanoma [3, 5].

Its origin remains controversial: the original germ was described [6], but the study by electron microscopy showed the presence of epithelial cells [7]. Moreover, the absence of a specific immunohistochemical profile of this tumor is an independent entity. It can express the EMA, cytokeratin, vimentin, NSE, CD10, WT1, and p53.

A single case of small cell carcinoma of the ovary expressing CD34 has been described in the literature [8]. Currently there is no consensus for the treatment of small cell carcinoma of the ovary. Although at the early stage of the disease, where the tumor is unilateral, radical surgery with removal of the uterus is recommended. But, it is considered desirable to preserve the fertility of these young patients. Therefore, the conservative surgical treatment sparing the ovary and uterus could be seen in the early stages [9].

However, despite the poor prognosis of patients with advanced small cell carcinoma of hypercalcemic type of ovarian cancer, an aggressive approach with radical resection followed by chemotherapy is widely applied [10].

Patients who received adjuvant chemotherapy had better survival in FIGO stage IA compared with those who had only surgical treatment [11].

Several protocols have been used including those used in the treatment of malignant epithelial tumors of the ovary: CP, CAP, AMP (cyclophosphamide, doxorubicin with or without Cisplatin, and hexamethylmelamine), but none of these protocols have proved effective [8, 12, 13]. The same limited effect was observed with paclitaxel in combination with Cisplatin with advanced disease by analogy to other epithelial tumors of the ovary [14].

Moreover, the best result was obtained with BVP and BEP (Cisplatin, Vinblastine, or Bleomycin with Etoposide) used in the treatment of germ cell tumors [15] justifying the use of this protocol in our patient.

In case of stage III disease, improved efficiency was observed using a scheme consisting of postoperative chemotherapy-based BEP followed by consolidation chemotherapy consisting of the following products: Vincristine, Actinomycin, and Cyclophosphamide [3, 16].

In the literature, most patients who had a better survival had received radiotherapy after or concurrent with chemotherapy: radiotherapy of any abdominal or pelvic radiotherapy and para-aortic [1, 3, 15].

In the study of Harrison et al. [15], five of seven patients received radiotherapy after or simultaneously with chemotherapy survived longer. However, due to the small number of patients, the additional benefits of radiotherapy, in terms of survival, remain to be established. Young et al. [3] reported that good prognostic factors are the localized stage of tumor, being smaller than 10 cm, and the patient age above 30 years. Tumors without large contingent of cells are also associated with a significantly higher survival. For cons, the presence of hypercalcemia preoperatively is a pejorative criterion [3].

The small cell carcinoma of hypercalcemic type of the ovary is a very aggressive tumor with a poor prognosis. The overall survival rate is about 10% and reaches 30% when the tumor is stage IA [16]. In the series of Young, a better survival (1 to 13 years) for stage IA and for patients who had undergone radical surgery was reported.

The cases we report describe a tumor larger than 10 cm in a young woman whose age is less than 30 years, with continued early evolutionary changes rapidly explained to be fatal.

## 4. Conclusion

The hypercalcemic small cell carcinoma of the ovary is a rare disease of poor prognosis; because of the rarity of the tumor and in the absence of large series evaluating therapeutic strategies adapted, it is reasonable to propose at this time the same strategies as those for other surgical ovarian tumors and a specific chemotherapy.
